# Clinical training exposure and perceived stress among nursing students: Examining the moderating role of social support

**DOI:** 10.1371/journal.pone.0361336

**Published:** 2026-10-26

**Authors:** Majed M. Aljabri, Bandar S. Alharbi, Endale Alemayehu Ali

**Affiliations:** 1 Community and Psychiatric Mental Health Nursing Department, College of Nursing, King Saud University, Riyadh, Saudi Arabia; 2 Department of Public Health and Primary Care, KU Leuven, Leuven, Belgium; National Institutes of Health, University of the Philippines Manila / De La Salle University, PHILIPPINES

## Abstract

**Background:**

Psychological stress is highly prevalent among nursing students. The clinical exposure is a major factor contributing to psychological stress, but there is a lack of consistent evidence, particularly in the context of Middle Eastern countries. The aim of this study was to quantify the association between clinical exposure and perceived stress among nursing students, and to assess whether perceived social support moderates this relationship.

**Methods:**

We conducted a cross-sectional study among nursing students from King Saud University. The Perceived Stress Scale was used to measure psychological stress, and the Multidimensional Scale of Perceived Social Support was employed to measure perceived social support. The clinical exposure was quantified as the average number of hours spent per week. We employed multivariable linear regression to determine the association. The regression analysis was adjusted for age, gender, living arrangements, and employment status.

**Results:**

Higher perceived social support was significantly associated with lower perceived stress (β = −0.05, 95% CI −0.09 to −0.01). Clinical exposure was not significantly associated with perceived stress. Students living alone had significantly lower levels of perceived stress (β = −0.37, 95% CI −0.63 to −0.11), as did students working part-time (β = −0.34, 95% CI −0.54 to −0.13). The interaction model revealed that perceived social support was inversely associated with perceived stress levels (β = −0.14, 95% CI −0.23 to −0.04). A statistically significant interaction was observed among students with less than 10 hours of clinical exposure per week. This exploratory finding suggests that the association between perceived social support and stress may differ among students with limited clinical exposure. However, the finding was based on a single statistically significant interaction and should be considered preliminary and hypothesis-generating.

**Conclusions:**

Higher perceived social support was associated with lower levels of perceived stress among nursing students. Interventions aimed at enhancing the social support system may be beneficial in reducing stress, especially during the early clinical exposure period.

## 1. Introduction

Psychological stress among university students is a well-documented global concern. The prevalence has been consistently reported across diverse educational settings [1]. Studies also emphasized that nursing students, in particular, experience high stress levels due to intensive coursework and clinical exposure responsibilities [2–4]. In addition, clinical education, which is essential for developing nursing competence, exposes students to complex healthcare environments, patient suffering, and high performance expectations, all of which can contribute to increased psychological stress [5,6]. The prevalence of stress among this population has been linked to poor performance, burnout, and dropout from nursing programs [7,8].

In several previous studies, clinical exposure, which is defined by the intensity and duration of clinical exposure, has been established as a determinant of stress among nursing students [4,9,10]. Increased duration of clinical exposure and workload, and balancing academic and training requirements, were established as factors that contribute to increased perceived stress levels among students [4]. The transactional model of stress and coping argues that stress is not only generated by external factors, but also by individuals’ perceptions of their ability to cope with external demands [11]. This should be reconsidered because the study setting is Saudi Arabia and the manuscript does not establish that the setting is resource-constrained. I recommend removing this phrase. However, not all students who experience similar clinical demands experience stress to a similar extent, indicating the presence of moderating or buffering factors. Perceived social support, which is the perception that one is loved, valued, and cared for by others and has a network of supportive social contacts, has been consistently shown to be a protective factor against psychological distress [12,13]. A study showed that in the context of nursing education, perceived social support from family, peers, and instructors has been shown to reduce stress, build resilience, and improve overall well-being [14]. Another study also found strong positive association between social support and psychological well-being among nursing students [15]. According to the stress-buffering hypothesis, social support reduces the effects of stressors by strengthening coping skills and decreasing perceived threats [13].

Although previous studies assessed the topic of stress and social support among nursing students, there is limited evidence on the burden of clinical exposure and its association with social support. Specifically, such studies are scarce in Middle Eastern countries. It is possible that cultural, educational, and healthcare system factors might influence both levels of stress and perceptions of available social support; hence, it is not possible to generalize results from studies conducted in Western culture and generalize them to Middle Eastern setting. Nursing students in Saudi Arabia have to cope with educational pressures as well as evolving professional demands in a dynamic healthcare system [16].

Therefore, we aimed to quantify the association between clinical exposure defined in terms of clinical hours per week and perceived stress levels among nursing students. In addition, our study further examined whether perceived social support defined in terms of scores on the Multidimensional Scale of Perceived Social Support (MSPSS) moderates this relationship. Using the theory of stress and coping, we hypothesized that higher levels of clinical exposure would be associated with higher levels of perceived stress and that perceived social support would moderate this association.

## 2. Method

### 2.1. Study design and data

We conducted a cross-sectional study among nursing students at King Saud University, Saudi Arabia. Data collection was conducted between 10 and 20 May 2026 during the regular academic semester. As the Perceived Stress Scale assesses stress experienced during the preceding month, responses may reflect academic and clinical demands occurring during this period.

All undergraduate and postgraduate nursing students enrolled at King Saud University during the study period were eligible to participate. We invited students to participate through a QR code displayed on university announcement boards. By scanning the QR code, students were directed to an online survey platform where they could review the study information and provide informed consent before participation. We excluded incomplete responses on key study variables from the analysis.

### 2.2. Outcome variable

Perceived stress was measured using the 10-item Perceived Stress Scale (PSS-10). This standard and validated tool was developed by Sheldon Cohen et al.[17]. The tool evaluates the degree to which individuals perceive situations in their lives as stressful during the previous month. We calculated total scores by summing item responses, with higher scores indicating greater perceived stress.

### 2.3. Predictor and moderator variables

Students were asked about their clinical exposure based on the number of hours they were required to devote to clinical exposure, apart from academic coursework. Clinical exposure was measured as the average number of clinical training hours per week and categorized into four groups: none, < 10 hours, 10–20 hours, and >20 hours. We assessed the moderating effects of perceived social support using the Multidimensional Scale of Perceived Social Support (MSPSS), consisting of 12 items measuring support from family, friends, and significant others. The original items were adopted from the developer D. Zimet et al.[12]. We computed the overall score as the mean of all items, with higher scores indicating greater perceived social support.

### 2.4. Covariates

We adjusted for several sociodemographic and contextual variables in the model

- Age group (18–20, 21–23, ≥ 24 years)
- Gender (female, male)
- Living arrangement (with family, with friends, alone, on-campus housing)
- Employment status (not working, part-time, full-time). The survey captured employment status irrespective of the type of occupation and did not specifically assess nursing-related employment.

### 2.5. Reliability assessment

Cronbach’s alpha coefficients were used to evaluate internal consistency of the MSPSS and PSS-10 scales.

### 2.6. Statistical analysis

We used multiple linear regression to quantify the association between clinical exposure and perceived stress. Perceived stress (PSS score) was the dependent variable. Prior to regression analysis, the PSS-10 total score was transformed using the natural logarithm of the PSS-10 score plus one, log(PSS + 1), because the outcome included zero values. This transformation allowed all observations, including participants with a PSS-10 score of zero, to be retained in the regression analyses. Descriptive statistics are presented using the original PSS-10 scale, whereas regression coefficients and predicted values are based on the log-transformed outcome. Clinical exposure was the primary independent variable, and perceived social support was evaluated as both an independent variable and a moderator through the inclusion of interaction terms. Age group, gender, living arrangement, and employment status were included as covariates based on their potential associations with stress reported in previous literature. To assess the moderating effect of social support, we included an interaction term between clinical exposure and MSPSS score. To facilitate interpretation of interaction effects and reduce potential multicollinearity between main effects and interaction terms, the MSPSS score was mean-centered prior to inclusion in the regression models. The centered MSPSS variable was used in all multivariable analyses. Model assumptions were evaluated through visual inspection of residual diagnostic plots, including residuals versus fitted values, normal Q-Q plots, scale-location plots, and residuals versus leverage plots. Multicollinearity was assessed using variance inflation factors (VIF), with values below 5 indicating acceptable levels. We used Generalized VIFs (GVIF) for categorical variables with more than two levels, and adjusted GVIF^(1/(2*df)) values are reported to facilitate interpretation.

Predicted values from the interaction model were used to visualize the moderating effect of social support on the relationship between clinical exposure and perceived stress.

### 2.7. Ethical approval

This study was conducted in accordance with the principles of the Declaration of Helsinki. Ethical approval was obtained from the Research Ethics Committee of King Saud University (Approval No. KSU-HE-26–0104) on 10 February 2026. Before accessing the survey, participants were provided with information regarding the study objectives, procedures, voluntary nature of participation, confidentiality, and their right to withdraw. Electronic informed consent was obtained from all participants prior to participation by requiring them to indicate their consent on the online survey platform before proceeding to the questionnaire.

We conducted the analysis in R version 4.2.1 [18].

## 3. Results

The distribution of perceived stress scores across categories of weekly clinical exposure and the association between perceived social support and stress among nursing students are presented in Fig 1 (Panel A and Panel B, respectively). We observed that the median stress scores are relatively similar across all clinical exposure groups. Although there are minor variations, the interquartile ranges overlap substantially across groups. There are a few outliers in each category, indicating individual variability in stress experiences, but there is no clear pattern of increasing stress with higher clinical exposure. The negative slope of the fitted regression line shows that higher levels of perceived social support are associated with lower perceived stress among students. With some variation in the individual variation, the overall downward trend suggests that social support may play a protective role in reducing stress.

**Fig 1:**
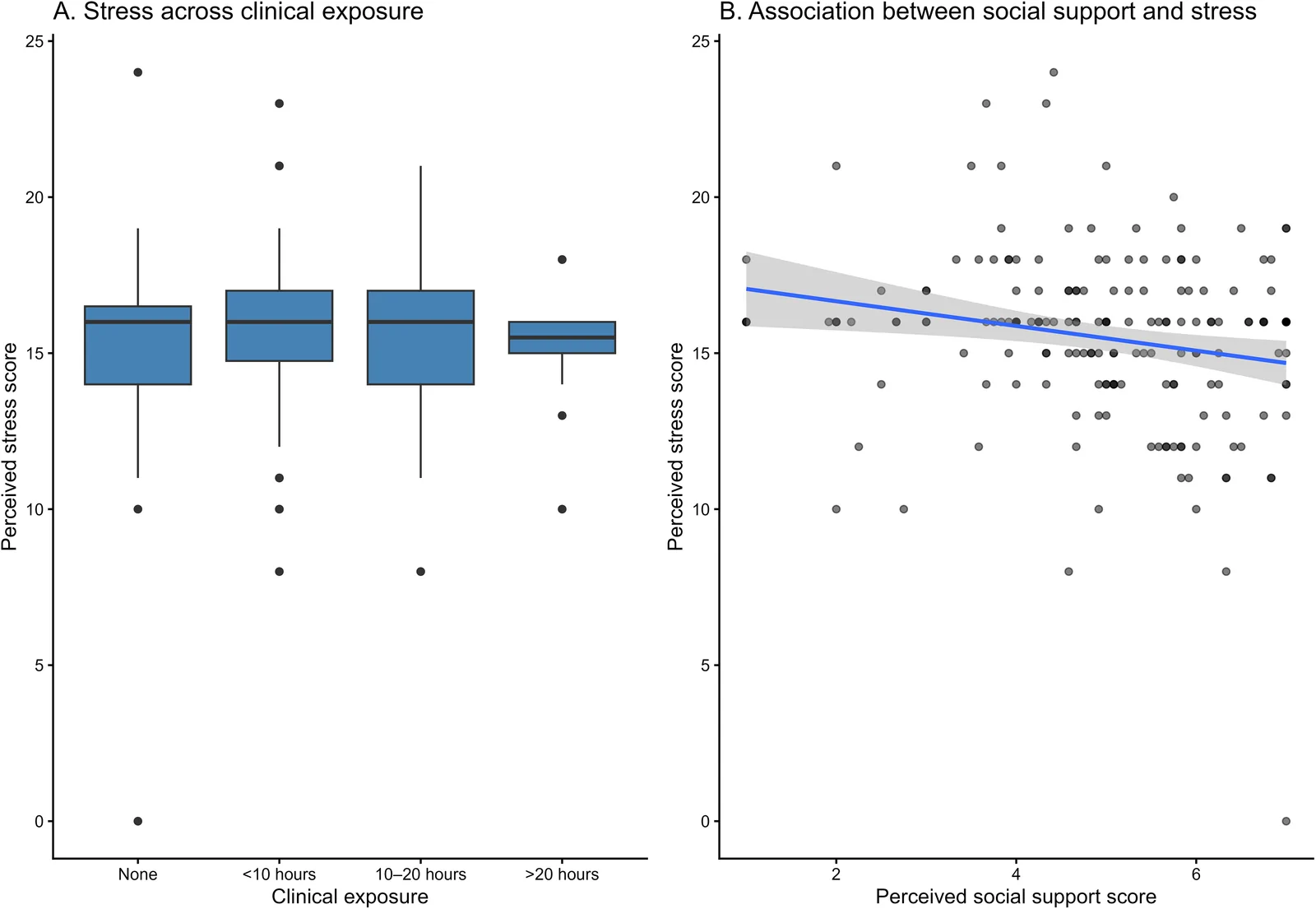
Distribution of perceived stress scores by (A) clinical exposure categories and (B) association between perceived social support and stress among nursing students.

The distribution of participants’ characteristics, including demographic, academic, and contextual characteristics, is provided in the supplementary material (S1 Fig in S1 File). Of the total 182 respondents, most participants were aged21–23 years (56%), followed by those aged 18–20 years (29%). The distribution by gender was relatively balanced, with 52% males and 48% females. Most participants were enrolled in undergraduate programs (88%), whereas 12% were postgraduate students. About 46% of the respondents do less than 10 hours of clinical exposure per week. Additionally, 23% (n = 41) reported 10–20 hours, 21% (n = 39) reported no clinical exposure, and a smaller proportion (9.9%, n = 18) reported more than 20 hours of clinical exposure per week. Students with no clinical exposure represented participants who were not engaged in clinical exposure during the study period. The majority of participants (79%) live with their families, and most students (80%) are not employed.

The mean perceived stress score among participants varied across levels of clinical exposure. Students with no clinical exposure had a mean PSS score of 17.4 (SD = 6.3), while those with less than 10 hours of clinical exposure per week had a mean score of 16.8 (SD = 6.8). Mean stress scores were slightly higher among students with 10–20 hours of clinical exposure (mean = 18.8, SD = 4.4) and among those with more than 20 hours of exposure (mean = 19.1, SD = 3.1).

In our study, both MSPSS and PSS-10 showed excellent reliability with corresponding Cronbach’s α of 0.96 and 0.89, respectively. This means the scales’ measurements were suitable for examining perceived social support and perceived stress.

Table 1 presents the results of a multivariable linear regression model examining factors associated with perceived stress among students. Because the PSS-10 score was log-transformed before regression, the regression coefficients represent associations with the natural logarithm of the PSS-10 score. We found a statistically significant association of perceived social support and stress level (β = −0.05, 95% CI: −0.09 to −0.01, p = 0.018), after adjusting for demographic and contextual variables. There is no significant association between clinical exposure hours and perceived stress. Compared with students reporting no clinical exposure, those with <10 hours (β = 0.09, 95% CI: −0.08 to 0.25), 10–20 hours (β = 0.08, 95% CI: −0.12 to 0.27), and >20 hours (β = 0.04, 95% CI: −0.28 to 0.36) showed slight increase, albeit not statistically significant. Our findings showed that compared to students living with their families, those living alone are significantly associated with lower stress (β = −0.37, 95% CI: −0.63 to −0.11). No significant differences were observed for students living with friends or in on-campus housing. Students working part-time reported significantly lower stress compared with those not working (β = −0.34, 95% CI: −0.54 to −0.13, p = 0.002), whereas full-time employment was not significantly associated with stress. We did not find significant association of Age group and gender with perceived stress. The diagnostic plots indicated minor departures from normality and homoscedasticity, with a small number of potential outlying observations (S2 Fig in S1 File). However, no influential observations exceeding conventional Cook's distance thresholds were identified.

**Table 1:** Association between predictors and perceived stress.

Characteristic	β (95% CI)	p-value
Clinical exposure (hours/week)		
None	Reference	—
<10 hours	0.09 (−0.08, 0.25)	0.300
10–20 hours	0.08 (−0.12, 0.27)	0.400
>20 hours	0.04 (−0.28, 0.36)	0.800
Perceived social support (MSPSS, centered)	−0.05 (−0.09, −0.01)	0.018
Age group (years)		
18–20	Reference	—
21–23	0.05 (−0.10, 0.19)	0.500
≥24	0.16 (−0.12, 0.43)	0.300
Gender		
Female	Reference	—
Male	0.02 (−0.12, 0.16)	0.800
Living arrangement		
With family	Reference	—
With friends	0.07 (−0.24, 0.39)	0.600
Alone	−0.37 (−0.63, −0.11)	0.005
On-campus housing	−0.01 (−0.20, 0.19)	0.930
Employment status		
Not working	Reference	—
Part-time	−0.34 (−0.54, −0.13)	0.002
Full-time	−0.09 (−0.34, 0.16)	0.500

Note: β coefficients represent adjusted estimates from the multivariable linear regression model. MSPSS scores were mean-centered before inclusion in the regression model. The dependent variable in all regression models was the log-transformed PSS score.

Table 2 presents the results of a multivariable linear regression model examining the interaction between clinical exposure and perceived social support in relation to perceived stress. We found that higher perceived social support remained significantly associated with lower perceived stress (β = −0.14, 95% CI: −0.23 to −0.04). This means that students with greater social support tended to experience lower levels of stress.

**Table 2:** Interaction between perceived social support and clinical exposure in predicting log-transformed PSS-10 scores.

Characteristic	β (95% CI)	p-value
Clinical exposure (hours/week)		
None	Reference	—
<10 hours	0.06 (−0.11, 0.22)	0.500
10–20 hours	0.05 (−0.15, 0.25)	0.600
>20 hours	0.01 (−0.31, 0.33)	0.920
Perceived social support (MSPSS, centered)	−0.14 (−0.23, −0.04)	0.005
Living arrangement		
With family	Reference	—
With friends	0.05 (−0.27, 0.38)	0.700
Alone	−0.31 (−0.58, −0.05)	0.021
On-campus housing	0.00 (−0.20, 0.19)	0.965
Age group (years)		
18–20	Reference	—
21–23	0.07 (−0.08, 0.22)	0.400
≥24	0.19 (−0.10, 0.47)	0.200
Gender		
Female	Reference	—
Male	0.03 (−0.11, 0.17)	0.700
Employment status		
Not working	Reference	—
Part-time	−0.35 (−0.56, −0.14)	0.001
Full-time	−0.08 (−0.33, 0.17)	0.500
Interaction: Clinical exposure × MSPSS		
<10 hours × MSPSS	0.11 (0.00, 0.22)	0.043
10–20 hours × MSPSS	0.07 (−0.07, 0.21)	0.314
>20 hours × MSPSS	0.16 (−0.16, 0.47)	0.322

Note: β coefficients represent adjusted estimates from the multivariable linear regression model. MSPSS scores were mean-centered before inclusion in the regression model. The dependent variable in all regression models was the log-transformed PSS score.

We did not find a significant association between clinical hours and perceived stress. However, compared with students with no clinical responsibility, those with <10 hours (β = 0.06, 95% CI: −0.11 to 0.22), 10–20 hours (β = 0.05, 95% CI: −0.15 to 0.25), and >20 hours (β = 0.01, 95% CI: −0.31 to 0.33) showed increased stress, though the association did not reach statistical significance.

However, we observed a significant interaction between social support and clinical exposure among students with <10 hours of clinical exposure (β = 0.11, 95% CI: 0.00–0.22). This finding suggests that the association between social support and stress differed for students with limited clinical exposure compared with those without clinical exposure. Specifically, the stress-reducing effect of social support was attenuated among students with <10 hours of clinical exposure. No statistically significant interactions were observed for students with 10–20 hours or more than 20 hours of clinical exposure.

A strong association was found between students living alone and lower perceived stress compared to those living with family (β = −0.31, 95% CI: −0.58 to −0.05). Similarly, part-time employment was associated with lower stress levels, compared with students who were not working (β = −0.35, 95% CI: −0.56 to −0.14, p = 0.001). There was no evidence of association of age group, gender, and full-time employment with perceived stress.

The findings reveal that there is no indication of problematic multicollinearity among the independent variables (Supplementary, S1 Table in S1 File). All the adjusted GVIF values, were much lower than the critical value of 5. This reveals that the regression coefficient estimates are not adversely impacted by the multicollinearity. Though the GVIF value for the interaction effect between clinical exposure and perceived social support was higher, the adjusted value was within the acceptable limits.

Fig 2 illustrates the predicted relationship between perceived social support and perceived stress across categories of clinical exposure. Consistent with the regression results, the association between social support and stress differed significantly only among students with less than 10 hours of clinical exposure per week. This finding suggests that the inverse association between perceived social support and perceived stress may be less pronounced in this group compared with students reporting no clinical exposure. Given the exploratory nature of the analysis and the modest statistical evidence supporting the interaction, this finding should be interpreted cautiously and requires confirmation in larger studies. No statistically significant interactions were observed among students with 10–20 hours or more than 20 hours of clinical exposure per week; therefore, differences in the slopes for these groups are presented for descriptive purposes only and should not be interpreted as evidence of effect modification.

**Fig 2:**
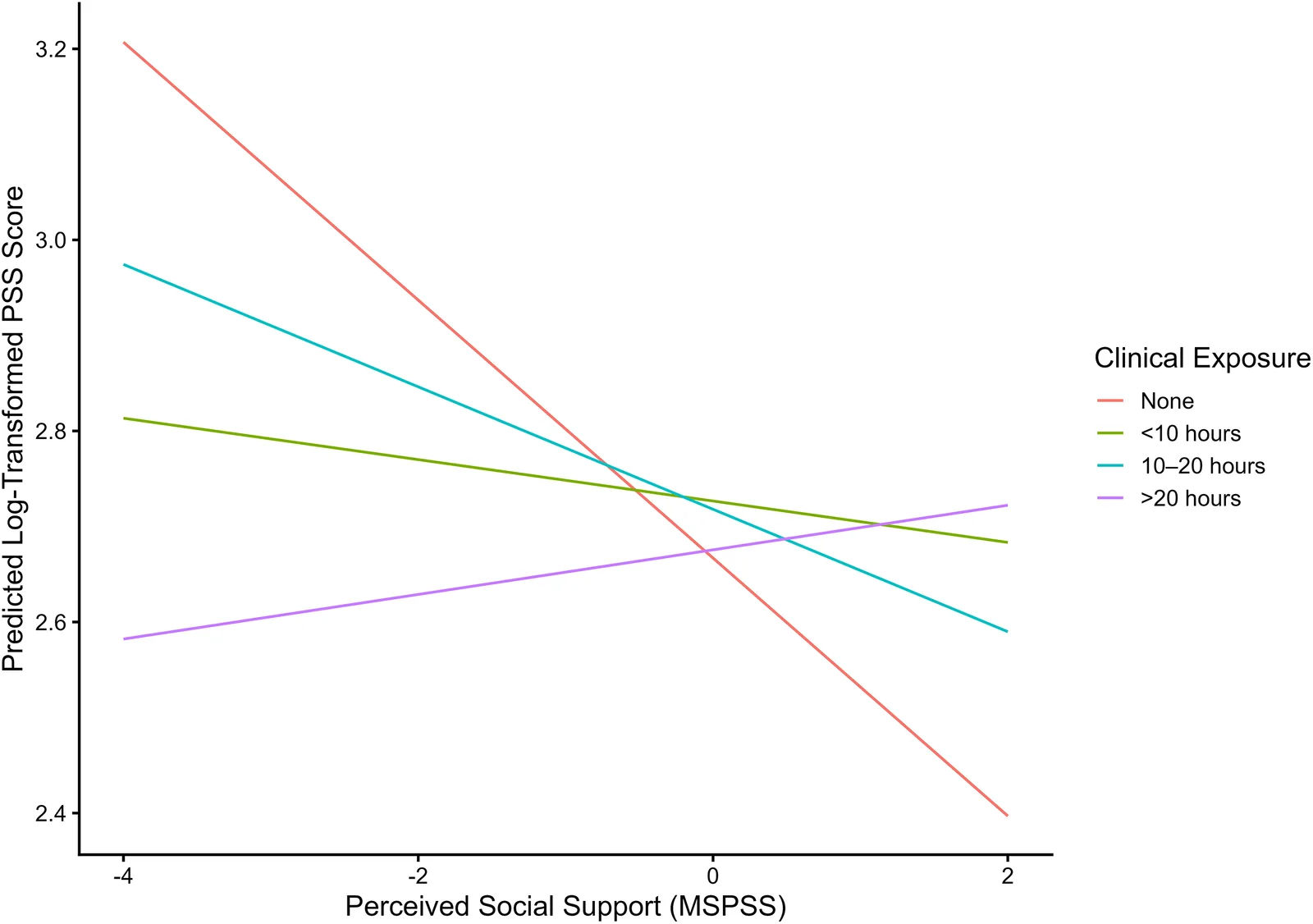
Interaction between perceived social support and clinical exposure in predicting log-transformed PSS-10 scores among nursing students. Note: Predicted values were derived from the adjusted interaction model. MSPSS scores were mean-centered before inclusion in the model. The figure is intended to visualize the regression results presented in Table 2.

## 4. Discussion

### 4.1. Principal findings

This study aimed to determine the association between clinical exposure and perceived social support and perceived stress levels among nursing students, especially in relation to the moderating effect of social support. Multivariable linear regression and interaction modeling analysis showed that higher levels of social support are associated with lower levels of perceived stress. However, clinical exposure hours were not significantly associated with perceived stress. In addition, a statistically significant interaction was observed among students reporting less than 10 hours of clinical exposure per week, suggesting that the association between social support and stress may differ in this group. Given the exploratory nature of the analysis and the modest statistical evidence supporting this interaction, the finding should be interpreted cautiously. Notably, being alone and having a part-time job are associated with low levels of stress.

### 4.2. Comparison with existing studies

The inverse association of perceived social support and stress, in our finding, is consistent with several previous studies. For example, a quantitative study on first year undergraduate students from the Ireland found strong association between social support and lower levels stress [19]. A study found that perceived social support plays important role on bullying behaviors of nursing students which might be linked to the stress due to clinical exposure and coursework that leads to stress [20]. Our finding further support the stress-buffering hypothesis by Cohen and Wills (1985) that states that social support reduces the harmful impacts of stressors by strengthening coping ability and reducing perceived threats [13]. The actual mechanism of social support is complex and may involve several pathways, such as emotional support, instrumental support, and cognitive support, which may be particularly important for coping with stress and challenges faced by nursing students. The MSPSS used in our study assesses perceived support from three key sources: family, friends, and significant others. In return, these support networks may provide important psychological and social resources that help students cope with stress and maintain well-being during their training.

The association between part-time employment and lower perceived stress should be interpreted cautiously. Additional since most students engaged in part-time employment were undergraduate students, the association is unlikely to be explained solely by postgraduate status. Nevertheless, students who undertake part-time work may differ from their peers in terms of motivation, self-efficacy, professional experience, or time-management skills. These factors were not measured in the present study and may partially account for the observed relationship.

Contrary to our initial hypothesis and previous findings, our study did not reveal a statistically significant association between clinical exposure and perceived stress. This result is inconsistent with previous findings. For example, a study in Saudi Arabia, found that greater clinical exposure and long hours in the clinical environment were significantly related to increased levels of stress in nursing students [4]. Several possible explanations may account for this discrepancy. It is possible that measurement differences contribute to the discrepancy. For example, several previous studies have used composite scales to measure clinical stress, focusing on qualitative aspects of clinical experiences, including fear of making mistakes and instructor/patient care complexity [21]. By contrast, our study used a quantitative approach to measure clinical exposure in terms of hours spent per week. This approach does not capture subjective experience during clinical training. Adaptation and coping mechanisms may also play a role. For example, it is possible that students who were exposed to higher clinical hours developed a coping mechanism for dealing with clinical experiences, thereby minimizing perceived clinical stress. The categorization of clinical exposure into broad time intervals may also have reduced our ability to detect differences between exposure groups. Our study assessed the interaction between clinical exposure and social support. A statistically significant interaction was observed only among students reporting less than 10 hours of clinical exposure per week. This finding suggests that the inverse association between social support and perceived stress may be less pronounced in this group than among students reporting no clinical exposure. For example, a cross-sectional study by Zhang et al. (2022) conducted among Chinese nursing students reported that social support mediated the association between stressors and self-regulatory fatigue, and the buffering effect of social support increased at higher levels of stressors [22]. Although our findings provide preliminary evidence that the association between social support and perceived stress may differ according to levels of clinical exposure, the interaction analysis was exploratory in nature and the study was not specifically designed or powered to detect interaction effects. Therefore, this finding should be considered hypothesis-generating and interpreted with caution until confirmed in larger studies specifically designed to assess effect modification. We also observed an association between living alone and lower perceived stress. This result contradicts some studies indicating that family support reduces stress [14,23]. However, the result might be attributed to specific cultural and environmental factors. Living with family members might imply some burden, which may contribute to stress. On the other hand, students living alone may have greater autonomy, which might help them manage their lives effectively, may facilitate stress management. We observed that part-time work was associated with less stress, whereas full-time work was not significantly associated. The result seems to be counterintuitive, as one might expect work to be an extra source of stress. A cross-sectional study among Australian nursing students observed that hours paid work were negatively associated with academic performance, which might increase stress [24]. The finding that students living alone reported lower stress than those living with their families warrants consideration within the Saudi sociocultural context. Family support is traditionally regarded as an important protective factor in Saudi society, and previous studies have often linked family connectedness with better psychological well-being. However, living with family may also involve substantial social obligations, household responsibilities, and academic expectations that can create additional sources of stress. Nursing students often face demanding schedules due to coursework and clinical exposure, and balancing these responsibilities with family commitments may increase perceived stress. Conversely, students living alone may benefit from greater autonomy in managing their daily routines, study schedules, sleep patterns, and clinical placements. Therefore, the observed association may reflect both environmental and individual factors. Given the cross-sectional design, further research is needed to explore the mechanisms underlying this relationship.

### 4.3. Strengths and limitations

Our study has several strengths. It utilizes widely used instruments to assess perceived stress and perceived social support constructs, i.e., Perceived Stress Scale (PSS) and Multidimensional Scale of Perceived Social Support (MSPSS). The reliability of these tools has already been established through various studies, and the high internal consistency of the results only adds to the reliability of the study. In addition, the application of multivariable linear regression enabled the quantification of independent associations between clinical exposure, social support, and perceived stress while adjusting for relevant demographic and contextual factors. The interaction analysis also allowed us to explore the potential moderating role of social support, providing a more comprehensive understanding of factors associated with stress among nursing students. The inclusion of various dimensions of the living and working environment of the students, i.e., living arrangement and employment status, also provided additional contextual information about factors associated with perceived stress.

Several limitations should also be acknowledged. First, the study design does not allow us to understand the causality of the results which have been obtained. In this case, the direction of the association between social support and stress cannot be established, which means the students may experience higher levels of stress, which can then affect their perceptions of seeking higher levels of support as well. Moreover, the self-reported data may lead to bias, which may introduce recall and social desirability bias. Because the survey was distributed electronically and information on the total number of students who received or accessed the survey was unavailable, a response rate could not be calculated. Consequently, the representativeness of the sample could not be formally assessed, and selection bias cannot be ruled out. The relatively homogeneous study population may have limited variation in some exposures and reduced the precision of subgroup estimates. In addition, although some relevant covariates were included, the possibility of confounding cannot be excluded, especially for unmeasured variables such as personality, coping styles, academic achievement, and prior mental health status. The study was exploratory in nature and was not specifically designed or powered to detect interaction effects. Because interaction analyses generally require substantially larger sample sizes than analyses of main effects, the observed interaction between social support and clinical exposure should be interpreted with caution. The relatively modest effect size and borderline statistical significance suggest that this finding should be considered hypothesis-generating and warrants replication in larger studies specifically designed to assess effect modification. Some categories of living arrangement contained relatively small numbers of participants compared with students living with their families. Consequently, estimates for these groups may be associated with greater statistical uncertainty and should be interpreted with caution. The employment variable captured only employment status and did not distinguish between nursing-related and non-nursing-related work. Consequently, students classified as employed may have had substantially different professional responsibilities and work experiences. In particular, some postgraduate students may have been engaged in professional nursing practice, whereas undergraduate students may have held part-time jobs unrelated to healthcare.

## 5. Conclusion

The findings of this study showed that higher perceived social support was associated with lower perceived stress among nursing students, while the clinical was not strongly associated with perceived stress after adjustment. Although a statistically significant interaction was observed among students with less than 10 hours of clinical exposure per week, this finding should be considered exploratory and hypothesis-generating. Further studies with larger sample sizes are needed to determine whether the association between perceived social support and perceived stress varies across levels of clinical exposure.

## Supporting information

S1 File Supplementary materials for the study “Clinical Training Exposure and Perceived Stress Among Nursing Students: The Moderating Role of Social Support. ” This file contains Table S1, assessment of multicollinearity using generalized variance inflation factors; Figure S1, distribution of sociodemographic and clinical characteristics of the study participants; and Figure S2, diagnostic plots for assessment of linear regression assumptions.
(DOCX)

## Abbreviations

β: Regression coefficient
CI: Confidence Interval
PSS: Perceived Stress Scale
MSPSS: Multidimensional Scale of Perceived Social Support
GVIF: Generalized Variance Inflation Factors
