# Supplementary material for: Clinical training exposure and perceived stress among nursing students: Examining the moderating role of social support
Source: PLoS One. 2026 Oct 26;21(10):e0361336. doi: 10.1371/journal.pone.0361336 (PMC13704807; doi:10.1371/journal.pone.0361336)
Supplement: Supplementary file 1 [file pone.0361336.s001.docx]

**Supplementary Materials**

**Clinical Training Exposure and Perceived Stress Among Nursing Students: The Moderating Role of Social Support**

Majed M. Aljabri^1*^, Bandar S. Alharbi^1^, Endale Alemayehu Ali^2^*

^1^Community and Psychiatric Mental Health Nursing Department, College of Nursing, King Saud University, Riyadh 12375, Saudi Arabia

^2^Department of Public Health and Primary Care, KU Leuven, Kapucijnenvoer 33, 3000 Leuven, Belgium

^*^ Shared corresponding author (Email: [amajad@ksu.edu.sa](mailto:amajad@ksu.edu.sa); [endalestat@gmail.com](mailto:endalestat@gmail.com))

S1 Table. Assessment of multicollinearity using generalized variance inflation factors (GVIF)

| Variable | GVIF | Df | GVIF^(1/(2×Df)) |
| --- | --- | --- | --- |
| Clinical exposure (hours/week) | 2.981 | 3 | 1.200 |
| Perceived social support (MSPSS) | 5.495 | 1 | 2.344 |
| Living arrangement | 1.613 | 3 | 1.083 |
| Age group | 3.210 | 2 | 1.339 |
| Gender | 1.516 | 1 | 1.231 |
| Employment status | 1.868 | 2 | 1.169 |
| Clinical exposure × MSPSS | 6.727 | 3 | 1.374 |

 S1 Figure: Distribution of sociodemographic and clinical characteristics of the study participants.

S2 Figure. Diagnostic plots for assessment of linear regression assumptions.
